# Evaluating the Impact of a Digital Nutrition Platform on Cholesterol Levels in Users With Dyslipidemia: Longitudinal Study

**DOI:** 10.2196/28392

**Published:** 2021-06-10

**Authors:** Emily A Hu, Jared Scharen, Viet Nguyen, Jason Langheier

**Affiliations:** 1 Zipongo Inc DBA Foodsmart San Francisco, CA United States

**Keywords:** dyslipidemia, hyperlipidemia, lipids, cholesterol, digital, nutrition, meal planning, food environment, food ordering, food purchasing

## Abstract

**Background:**

A strong association exists between consuming a healthy diet and lowering cholesterol levels among individuals with high cholesterol. However, implementing and sustaining a healthy diet in the real world is a major challenge. Digital technologies are at the forefront of changing dietary behavior on a massive scale, as they can reach broad populations. There is a lack of evidence that has examined the benefit of a digital nutrition intervention, especially one that incorporates nutrition education, meal planning, and food ordering, on cholesterol levels among individuals with dyslipidemia.

**Objective:**

The aim of this observational longitudinal study was to examine the characteristics of people with dyslipidemia, determine how their status changed over time, and evaluate the changes in total cholesterol, high-density lipoprotein cholesterol (HDL-C), low-density lipoprotein cholesterol (LDL-C), non-HDL-C, and triglycerides among individuals with elevated lipids who used Foodsmart, a digital nutrition platform that integrates education, meal planning, and food ordering.

**Methods:**

We included 653 adults who used Foodsmart between January 2015 and February 2021, and reported a lipid marker twice. Participants self-reported age, gender, weight, and usual dietary intake in a 53-item food frequency questionnaire, and lipid values could be provided at any time. Dyslipidemia was defined as total cholesterol ≥200 mg/dL, HDL-C ≤40 mg/dL, LDL-C ≥130 mg/dL, or triglycerides ≥150 mg/dL. We retrospectively analyzed distributions of user characteristics and their associations with the likelihood of returning to normal lipid levels. We calculated the mean changes and percent changes in lipid markers among users with elevated lipids.

**Results:**

In our total sample, 54.1% (353/653) of participants had dyslipidemia at baseline. Participants with dyslipidemia at baseline were more likely to be older, be male, and have a higher weight and BMI compared with participants who had normal lipid levels. We found that 36.3% (128/353) of participants who had dyslipidemia at baseline improved their lipid levels to normal by the end of follow-up. Using multivariate logistic regression, we found that baseline obesity (odds ratio [OR] 2.57, 95% CI 1.25-5.29; *P*=.01) and Nutriscore (OR 1.04, 95% CI 1.00-1.09; *P*=.04) were directly associated with achieving normal lipid levels. Participants with elevated lipid levels saw improvements as follows: HDL-C increased by 38.5%, total cholesterol decreased by 6.8%, cholesterol ratio decreased by 20.9%, LDL-C decreased by 12.9%, non-HDL-C decreased by 7.8%, and triglycerides decreased by 10.8%.

**Conclusions:**

This study characterized users of the Foodsmart platform who had dyslipidemia and found that users with elevated lipid levels showed improvements in the levels over time.

## Introduction

Cardiovascular disease (CVD) is the leading cause of morbidity and mortality in the United States and globally [1]. The annual estimated cost of CVD in the United States is over US $200 billion in health care services, medications, and lost productivity. Dyslipidemia has been established as a strong risk factor for CVD. It has been estimated that one in three adults in the United States has dyslipidemia [2]. Dyslipidemia refers to elevated total cholesterol, low-density lipoprotein cholesterol (LDL-C), or triglycerides, or low levels of high-density lipoprotein cholesterol (HDL-C) [3]. While this condition can be due to genetic factors, it is usually associated with unhealthy lifestyle behaviors such as poor diet and physical inactivity.

Atherogenic lipoproteins play an important role in the initiation and progression of atherosclerosis; therefore, maintaining optimal lipid levels is crucial for achieving ideal cardiovascular health [4]. LDL and other apolipoprotein B–containing lipoproteins slowly accumulate in the artery wall early in life, which eventually can result in large amounts of atherosclerotic plaque. This can lead to obstruction of blood flow, which could cause cardiovascular events such as acute coronary syndrome and myocardial infarction. Statins are the most commonly prescribed lipid-lowering drug in patients with dyslipidemia. Other LDL-lowering drugs include PCSK9 inhibitors, ezetimibe, and bile acid sequestrants [5]. Despite high awareness of abnormal lipid levels (>80%), statin use has been found to be low (37.6%) among adults with severe dyslipidemia [2]. Issues with nonadherence or unwillingness to take cholesterol-lowering medications pose obstacles to reducing CVD risk.

For years, guidelines have suggested dietary modification to be a crucial component in strategies to reduce CVD risk [5,6]. Diet has been shown to have a major impact on lipid levels and CVD [7,8]. Studies have suggested that nuts, plants, and fiber-rich foods may reduce LDL-C levels [3]. Additionally, a dietary pattern low in saturated fats, low in refined carbohydrates, and rich in unsaturated fatty acids and proteins has been shown to be successful in reducing plasma LDL-C levels [9,10]. Despite the strong associations between dietary changes and cholesterol levels, many patients fail to adopt a healthy dietary pattern or make lasting changes. Thus, adoption and sustainability of a healthy diet are critical issues.

Many barriers to adopting and sustaining a healthy dietary pattern exist, such as time, cost, accessibility, and knowledge. Foodsmart is a digital nutrition and meal planning platform that is designed to make healthier eating achievable and sustainable among the general population, and it addresses the most common barriers to eating well. Foodsmart uses a multipronged approach including educating individuals on how to eat healthy, leveraging the food frequency questionnaire, recommending personalized healthy recipes based on food preferences, and automating grocery list creation and online grocery purchasing, all while tracking the individual’s improvements in biometrics. The platform has been found to be associated with at least 5% weight loss and has been shown to sustain weight loss over 3 years [11,12]. Previous research has suggested that by simply cooking at home rather than ordering food or eating out, diet quality improves [13]. Therefore, this digital platform that uses precision nutrition to encourage healthier eating and sustained practices has broad potential to improve important health markers such as lipid levels.

While many digital applications seek to improve eating behaviors and health outcomes, few studies have evaluated their effectiveness for changing lipid levels among users with dyslipidemia. The aim of this study was to examine the characteristics of users with dyslipidemia and evaluate the changes in lipid markers over time.

## Methods

### Study Sample

As of February 2021, 13,754 users of Foodsmart had entered a plausible value (defined later) for at least one lipid marker (total cholesterol, HDL-C, LDL-C, or triglycerides). Of those, 1445 users of Foodsmart had entered at least one lipid marker at two different time points. We excluded participants who reported their second lipid marker less than 1 month after their first report and those with implausible changes. Our final sample size was 653 participants who had at least two reports of at least one lipid marker.

### The Foodsmart Platform

Foodsmart is a digital nutrition platform that uses precision nutrition to create lasting behavior change through nutrition education and personalized recipe recommendations, and facilitates healthy eating through online grocery and food ordering integration. Rooted in behavior change theory, Foodsmart has two components, FoodSmart and FoodsMart, to help users access and engage with affordable, tasty, and healthy food.

The FoodSmart component emphasizes learning by helping the user understand how their typical eating behaviors compare to national targets and how to plan their meals for the week. Once users create their account, they are directed to the in-app Nutriquiz, a dietary assessment (based on the National Cancer Institute Diet History Questionnaire). Users report their usual dietary habits, and the quiz provides immediate and specific feedback on aspects of their diet to improve on. Over time, users can retake the Nutriquiz to track their progress on diet and biometrics. Based on the Nutriquiz results, personalized recipe recommendations are given to the user. The second component is FoodsMart, which focuses on altering the food purchasing environment to make healthier options the easier default path. This is achieved through personalized meal plan conversion to a grocery list and integrated online ordering and delivery of groceries, meal kits, and prepared foods, where food advertising paid for by food manufacturers is removed and replaced with nudges to healthier substitutions that align with user preferences and their personalized meal plan. Customized grocery discounts on healthier options help users save money and further nudge users to make healthier choices.

Foodsmart is available through health plans and employers and can be accessed via the web or the iOS or Android operating system.

### Measurements of Lipids and Weight

Users were given the option upon enrollment to input self-reported total cholesterol, HDL-C, LDL-C, triglycerides, weight, and height data. They could update their biometrics at any time during usage of the platform. All lipid markers were reported in mg/dL. Given the self-reported nature of lipids, we considered the following values as missing data: total cholesterol ≤65 or ≥750 mg/dL, HDL-C ≤10 or ≥120 mg/dL, LDL-C ≤30 or ≥200 mg/dL, and triglycerides ≤10 or ≥2000 mg/dL [14]. We only included people who reported a lipid measurement at least twice, and we used the first and last values. We defined the “end” value as the last value. We defined the following prespecified cutoffs as markers of dyslipidemia based on the National Cholesterol Education Program (NCEP) Adult Treatment Panel III (ATP III) classification of lipid profiles: total cholesterol ≥200 mg/dL, HDL-C ≤40 mg/dL, LDL-C ≥130 mg/dL, or triglycerides ≥150 mg/dL [15]. If any of these thresholds were met, the participant was considered to have dyslipidemia. The same method was applied to the end value to assess dyslipidemia at the end of follow-up. Changes in lipid markers were calculated by subtracting the first reported values from the end values. Percent change was calculated by dividing change in lipid values by the first lipid value. We also examined values of the cholesterol ratio (total cholesterol/HDL-C), with a threshold of ≥5 considered elevated.

Baseline BMI was calculated as first weight entry in kilograms divided by height in meters squared (kg/m^2^). We categorized participants by baseline BMI category as follows: normal BMI was defined as BMI <25 kg/m^2^, overweight was defined as BMI between 25 and 29.9 kg/m^2^, and obese was defined as BMI ≥30 kg/m^2^.

### Dietary Assessment

Participants self-reported their usual dietary intake in Foodsmart. Upon registration, users were prompted to fill out a dietary questionnaire called Nutriquiz, a 53-item food frequency questionnaire adapted from the National Cancer Institute Diet History Questionnaire [16]. Information on sex, age, weight, and usual frequency of dietary intake (fruits, vegetables, whole grains, proteins, carbohydrates, fats, fiber, sodium, and water) were ascertained in Nutriquiz. We calculated a score to assess overall diet quality (Nutriscore), which is based on the Alternative Healthy Eating Index-2010 and the Commonwealth Scientific and Industrial Research Organization Healthy Diet Score [17,18]. Participants were assigned a score from 0 to 10 (with 10 being optimal) for each of the following seven components: fruits, vegetables, protein ratio (white meat/vegetarian protein to red/processed meat), carbohydrate ratio (total fiber to total carbohydrate), fat ratio (polyunsaturated to saturated/trans fats), sodium, and hydration (percent of daily fluid goal). A total Nutriscore (possible scores ranging from 0 to 70) was calculated by summing the scores of the seven components. Change in the Nutriscore was calculated as the difference between a participant’s first and last Nutriscores.

### Statistical Analysis

We used descriptive analyses to examine the baseline demographic characteristics, lipid markers, and diet quality of the total study population and according to whether they had dyslipidemia at baseline. We reported categorical variables as number (percentage) and continuous variables as mean (SD). We used the chi-square test and analysis of variance to test for differences in categorical and continuous variables, respectively.

In order to better understand how the dyslipidemia status changed over time, we calculated the percent of participants by category of change in the dyslipidemia status from the beginning to the end of the program as follows: dyslipidemia to normal, normal to dyslipidemia, dyslipidemia to dyslipidemia, and normal to normal. Multivariate logistic regression was used to estimate the odds ratios (ORs) and 95% CIs of achieving normal lipid levels among participants with baseline dyslipidemia and was mutually adjusted for gender, age category, baseline BMI category, baseline Nutriscore, and change in Nutriscore (per 5 points).

Among participants who had elevated lipid levels, we calculated the mean start value, mean end value, and mean changes in total cholesterol, cholesterol ratio, HDL-C, LDL-C, non-HDL-C, and triglycerides. We used paired *t* tests to test whether the changes were statistically significant. Additionally, we calculated the mean percent change for each marker. To further explore the performance of LDL-C, we examined changes in LDL-C stratified by the category of baseline LDL-C (optimal: <100 mg/dL; above optimal: ≥100 and <130 mg/dL; and high: ≥130 mg/dL).

We considered a *P* value smaller than .05 to be significant for all tests. Stata version 16 (StataCorp) was used for all analyses.

The study was declared exempt from institutional review board oversight by the Pearl Institutional Review Board given the retrospective design of the study and the less than minimal risk to participants.

## Results

### Participant Characteristics

Baseline characteristics of the total study sample and those stratified by baseline dyslipidemia status are shown in Table 1. We found that 54.1% (353/653) of participants had dyslipidemia at baseline.

**Table 1:** Baseline characteristics of the total study sample and those stratified by baseline dyslipidemia status.

Characteristic		Total (N=653)			Normal (N=300)				Dyslipidemia (N=353)				*P* value^a^		
		Number of participants	Percentage or mean (SD)		Number of participants		Percentage or mean (SD)		Number of participants		Percentage or mean (SD)				
**Age (years)**													.11		
	<40	196		33%		100		36%		96		30%			
	40-59	306		51%		138		50%		168		52%			
	≥60	99		16%		38		14%		61		19%			
Female gender		346	53%		177		59%		169		48%		<.001		
Weight (kg)		649	79.4 (20.0)		299		75.3 (18.6)		350		83.0 (20.4)		<.001		
BMI (kg/m^2^)		649	27.3 (5.7)		299		26.1 (5.3)		350		28.3 (5.9)		<.001		
Total cholesterol (mg/dL)		632	180.1 (37.2)		289		163.1 (25.5)		343		194.4 (39.5)		<.001		
Cholesterol ratio^b^		608	3.6 (1.4)		276		2.8 (0.6)		332		4.3 (1.4)		<.001		
HDL-C^c^ (mg/dL)		623	54.6 (17.5)		284		61.2 (13.9)		339		49.0 (18.3)		<.001		
LDL-C^d^ (mg/dL)		606	102.0 (31.8)		275		88.0 (101.8)		331		113.7 (34.3)		<.001		
non-HDL-C (mg/dL)		608	125.1 (37.2)		276		101.8 (25.2)		332		144.5 (34.3)		<.001		
Triglycerides (mg/dL)		639	117.6 (37.2)		288		90.8 (30.8)		351		139.6 (77.2)		<.001		
Baseline Nutriscore (range 0-70)		351	34.3 (8.3)		168		34.6 (8.1)		183		34.1 (8.4)		.58		
Change in Nutriscore		389	2.4 (7.3)		181		2.4 (7.3)		208		2.4 (7.3)		.99		
Follow-up duration (months)		653	16.9 (11.3)		300		17.4 (11.5)		353		16.5 (11.2)		.33		

^a^Chi-square tests and analysis of variance were used to test differences for categorical and continuous variables, respectively.

^b^Cholesterol ratio was defined as total cholesterol/high-density lipoprotein cholesterol.

^c^HDL-C: high-density lipoprotein cholesterol.

^d^LDL-C: low-density lipoprotein cholesterol.

There were 653 participants included in the analysis, of which 306 were between 40 and 59 years old and 346 were female (Table 1). The mean BMI was 27.3 kg/m^2^, the mean baseline Nutriscore was 34.3 points, and the mean change in the Nutriscore was 2.4 points. The mean follow-up length was 16.9 months and ranged from 1 to 60 months. Compared to participants who did not have dyslipidemia, participants who had dyslipidemia were more likely to be in the 40-59 or ≥60 age categories, more likely to be male, and more likely to have a higher weight and BMI.

We calculated the percent of participants based on what category of dyslipidemia status change they were in. We categorized participants into four groups based on their dyslipidemia status at the beginning and end of their follow-up as follows: dyslipidemia to normal, normal to dyslipidemia, dyslipidemia to dyslipidemia, and normal to normal. We found that 19.6% (128/653) of participants had dyslipidemia in the beginning and achieved normal lipid levels by the end, 12.4% (81**/**653) developed dyslipidemia, 34.4% (225/653) had dyslipidemia and it did not change, and 33.5% (219/653) had normal lipid levels and they did not change. Among participants who had dyslipidemia at baseline, 36.3% (128/353) improved their lipid levels to normal by the end of follow-up.

In order to better understand what type of user was successful in achieving normal lipid levels, we examined the association between baseline characteristics and odds of achieving normal lipid levels in a multivariate logistic regression model (Table 2). Adjusting for all other variables, there was no significant association between gender or age and achieving normal lipid levels. Participants who were obese were 157% more likely to achieve normal lipid levels (OR 2.57, 95% CI 1.25-5.29; *P*=.01). Having a higher baseline Nutriscore (healthier diet quality) was also associated with higher odds of achieving normal lipid levels (OR 1.04, 95% CI 1.00-1.09; *P*=.04). Improvement in the Nutriscore was positively associated, though this was not statistically significant.

**Table 2:** Association between predictors and the likelihood of changing the dyslipidemia status to normal in multivariate logistic regression models.

Variable		Odds ratio (95% CI)		*P* value
Female		0.81 (0.45-1.46)		.49
**Age (years)**				
	<40	1 (reference)	N/A^a^	
	40-59	0.69 (0.33-1.43)	.32	
	≥60	2.13 (0.96-4.76)	.07	
**Baseline BMI category**				
	Normal	1 (reference)	N/A	
	Overweight	1.26 (0.63-2.55)	.51	
	Obese	2.57 (1.25-5.29)	.01	
Baseline Nutriscore		1.04 (1.00-1.09)		.04
Change in Nutriscore (per 5 points)		1.07 (0.86-1.33)		.56

^a^N/A: not applicable.

### Changes in Lipid Levels

Table 3 presents the mean start values, end values, and changes in lipid markers among participants who were classified as having elevated levels for each marker. The mean changes were as follows: total cholesterol, −16.4 (SD 34.4) mg/dL; cholesterol ratio, −1.5 (SD 1.7); HDL-C, 11.2 (SD 14.0) mg/dL; LDL-C, −20.6 (SD 30.1) mg/dL; non-HDL-C, −13.6 (SD 31.3) mg/dL; and triglycerides, −34.2 (SD 95.1) mg/dL. All changes were statistically significant (*P*<.001) using paired *t* tests.

**Table 3:** Changes in lipid levels among users with elevated lipid levels.

Variable	Number of participants	Start value, mean (SD)	End value, mean (SD)	Change, mean (SD)	*P* value^a^
Total cholesterol (mg/dL)	171	223.5 (21.1)	207.1 (31.8)	−16.4 (34.4)	<.001
Cholesterol ratio^b^	64	6.1 (1.3)	4.7 (0.9)	−1.5 (1.7)	<.001
HDL-C^c^ (mg/dL)	115	33.3 (5.9)	44.5 (13.2)	11.2 (14.0)	<.001
LDL-C^d^ (mg/dL)	90	150.7 (16.9)	130.1 (27.1)	−20.6 (30.1)	<.001
Non-HDL-C (mg/dL)	193	158.7 (23.0)	145.2 (30.7)	−13.6 (31.3)	<.001
Triglycerides (mg/dL)	107	213.3 (77.0)	179.1 (74.3)	−34.2 (95.1)	<.001

^a^*P* values were calculated using paired *t* tests.

^b^Cholesterol ratio was defined as total cholesterol/high-density lipoprotein cholesterol.

^c^HDL-C: high-density lipoprotein cholesterol.

^d^LDL-C: low-density lipoprotein cholesterol.

We determined the mean percent changes in total cholesterol, cholesterol ratio, HDL-C, LDL-C, non-HDL-C, and triglycerides among users with elevated lipid levels. The greatest percent change was in HDL-C (+38.5%), followed by cholesterol ratio (−20.9%), LDL-C (−12.9%), triglycerides (−10.8%), non-HDL-C (−7.8%), and total cholesterol (−6.8%).

To better understand how LDL-C changed according to baseline LDL-C, we examined the mean changes in LDL-C stratified by the category of baseline LDL-C (normal, slightly elevated, and moderate or highly elevated) (Table 4). We found that the greatest reduction in LDL-C was among people with high LDL-C. Among users who had normal baseline levels of LDL-C (<100 mg/dL), an increase in LDL-C was noted (mean 16.6, SD 31.3 mg/dL). Among users with slightly elevated LDL-C levels (≥100 and <130 mg/dL), there was a small decrease in LDL-C (mean −2.0, SD 23.0 mg/dL), and among users with moderate or highly elevated LDL-C levels (≥130 mg/dL), there was a large decrease in LDL-C (mean −20.6, SD 30.1 mg/dL).

**Table 4:** Changes in low-density lipoprotein cholesterol (LDL-C) levels according to the category of baseline LDL-C.

Category of baseline LDL-C^a^	Number of participants	Start LDL-C (mg/dL), mean (SD)	End LDL-C (mg/dL), mean (SD)	Change in LDL-C (mg/dL), mean (SD)
Normal (<100 mg/dL)	202	76.1 (16.9)	92.8 (30.8)	16.6 (31.3)
Slightly elevated (≥100 and <130 mg/dL)	148	113.5 (8.7)	111.5 (24.6)	−2.0 (23.1)
Moderate or highly elevated (≥130 mg/dL)	90	150.7 (16.9)	130.1 (27.1)	−20.6 (30.1)

^a^LDL-C: low-density lipoprotein cholesterol.

## Discussion

In our study, of 653 users who reported at least two lipid markers, we found that 54.1% (353/653) of participants had dyslipidemia at baseline, and of those, 36.3% (128/353) showed improvements in their lipid levels to normal by the end of follow-up. Participants with dyslipidemia at baseline were more likely to be older, be male, and have a higher weight and BMI. Baseline obesity and Nutriscore were associated with a higher likelihood of achieving normal lipid levels. Between the start and end of using the Foodsmart platform, total cholesterol, cholesterol ratio, LDL-C, and triglycerides all significantly decreased and HDL-C significantly increased. These findings suggest that usage of the Foodsmart platform may be associated with improvements in lipid markers, most likely through improved diet quality.

The results of this study support the findings of previous studies that found beneficial effects of dietary interventions on lipid levels among people with dyslipidemia. A meta-analysis of over 200 studies that examined the impact of dietary interventions on cholesterol levels found that a reduction in saturated fats and an increase in polyunsaturated fats were primary factors in lowering total cholesterol levels [19]. Another meta-analysis of 60 trials found that replacing trans fats with polyunsaturated fats was successful in improving blood lipids [20]. Increasing dietary soluble fiber has also been shown to decrease total and LDL cholesterol levels, although the effect was modest (5 mg/dL) [21]. In the landmark PREDIMED randomized controlled trial that tested the effect of two Mediterranean-style dietary patterns against a low-fat dietary pattern among participants at high risk of CVD, investigators found that just after 3 months, the cholesterol ratio decreased by 0.38 and 0.26 for a Mediterranean-style diet supplemented with extra-virgin olive oil and nuts, respectively [22].

Though the association between diet and cholesterol is strong and has been established for decades, implementing and sustaining behavior change in real life, especially with diet, is complex and challenging. It has been noted that physicians face many challenges in encouraging behavior change to improve lipid profiles and other CVD risk factors in patients [23]. A review found that patients who received dietary advice reduced total cholesterol levels by 6.2 mg/dL and LDL-C by 7.0 mg/dL, although there were no significant changes in HDL-C [24]. Another review found that dietitian advice was more successful in reducing cholesterol levels compared to physician advice (−9.7 mg/dL difference for total cholesterol), although it was not better than self-help materials [25]. There is sparse evidence of a digital intervention improving cholesterol levels, especially among commercial nutrition applications.

The annual per person expenditure related to dyslipidemia among people without CVD has been estimated to be about US $856 [26]. Annual national expenditure has been estimated to be US $23.1 billion. The majority of expenditures (59%-90%) were attributable to prescription medications, namely statins. High-intensity statins, such as rosuvastatin and atorvastatin, can lower LDL-C by 50% or more, while moderate-intensity statins lower LDL-C by 30%-49% and low-intensity statins lower LDL-C by less than 30% [5]. While statins have generally been regarded as safe, there are some side effects, such as myalgia, which is observed in 5%-20% of patients, leading to nonadherence [5]. Additionally, patients who are initially adherent to statin therapy may not continue to have long-term adherence for other reasons [27,28]. While PCSK9 inhibitors can result in further reductions in LDL-C and reduce risks of cardiovascular events, cost-effectiveness models have suggested that their high costs do not outweigh the potential benefits yet [5,29]. Given the high pharmaceutical costs of treating dyslipidemia, with questionable adherence, prevention and treatment of high cholesterol through a healthy diet can be considered an attractive option. Unfortunately, we did not have information on whether participants were on statins or other cholesterol-lowering medications. Therefore, we do not know whether participants had started taking medications before enrolling in Foodsmart, and if so, for how long they had been taking medications. Despite this lack of data, we sought to make a ballpark comparison of price points in lowering lipid levels between Foodsmart and prescription medications. Digital platforms represent an affordable alternative, as the mean annual cost for the Foodsmart platform is US $12.30 per eligible member as of 2021. Based on our analysis, the cost per 1% reduction in LDL-C was US $0.95 using Foodsmart. In the case of statins, on the low end, it would cost US $12.89 per 1% reduction in LDL-C for lovastatin (20 mg), and on the high end, it would cost US $77.50 per 1% reduction in LDL-C for rosuvastatin (10 mg) [30,31]. From these calculations and assuming that participants in the analysis were not on cholesterol-lowering medications, we estimate that a digital platform like Foodsmart is 93% to 99% more affordable per 1% reduction in LDL-C compared with standard statin treatment.

The present study has several limitations worth addressing. The first is that all lipid measurements were self-reported and were not validated. However, in a validation study among about 40,000 female health professionals in the Women’s Health Study, investigators found that the Spearman correlation coefficients between self-reported and blood samples for triglycerides and HDL-C were 0.57 and 0.63, respectively [32]. This suggests there is moderate correlation between self-reported and blood measures. Since users were not obligated to enter their cholesterol levels, it is more likely that people who did report their lipid levels had accurate reports (no guessing) and entered them into the app for the purpose of tracking. This also means that our participant pool may be subject to selection bias, as participants who were more aware of their cholesterol levels, possibly due to having a health condition, were more likely to be in our sample. Another limitation is that we unfortunately did not have participants’ medical history or medication use. Participants may have been on cholesterol-lowering medications, which could have contributed to improvements in lipid levels. We also did not know whether participants had a genetic condition related to high cholesterol levels. However, genetics-related cholesterol conditions are usually marked by extremely high cholesterol levels (>300 mg/dL) or triglyceride levels (>500 mg/dL) and therefore would not have been included in the analysis [2]. Future studies would benefit from collecting medical history and medication use to better elucidate and better understand these associations. In our analysis, we did not account for the frequency of engagement with the platform, which could have an effect on the associations. We also did not account for socioeconomic factors, such as baseline education, that are potential confounders. For future studies, we plan on assessing and incorporating engagement activities and education levels.

There are also many strengths of this study. Very few studies have demonstrated the real-life application of a digital intervention that changes a user’s meal planning and food ordering behaviors, and its effect on cholesterol levels. By leveraging our large database of users of the Foodsmart platform, we could evaluate real-world data to draw patterns and associations that provide insights into the utility of commercial digital applications. Additionally, many participants were enrolled for at least a year, allowing us to examine changes in lipids over a long time span. Few studies, especially randomized clinical trials, on digital applications have follow-up data for lipids after more than 2 years.

In conclusion, this is one of the first studies of this scale and duration to examine changes in lipids among individuals with dyslipidemia who were users of a digital nutrition platform with personalized dietary recommendations, as well as online meal planning, food ordering, and grocery discounts and incentives. Future studies are warranted to examine specific food components that are associated with lowering cholesterol levels, perform cost comparisons between pharmaceutical and digital interventions, and identify causal associations by comparing interventions to a control.

## Abbreviations

CVD: cardiovascular disease
HDL-C: high-density lipoprotein cholesterol
LDL-C: low-density lipoprotein cholesterol
OR: odds ratio
